# Artificial microRNAs against hypoxia-responsive small protein encoding genes reveal HRSP41 and 42 as regulators of development and stress tolerance

**DOI:** 10.1186/s12870-026-09666-5

**Published:** 2026-08-01

**Authors:** Mari-Cruz Castillo, Sandra Sánchez-Palomares, José León

**Affiliations:** Department of Development and Hormone Action, Instituto de Biología Molecular y Celular de Plantas (Consejo Superior de Investigaciones Científicas – Universidad Politécnica de Valencia), Valencia, 46022 Spain

**Keywords:** Small proteins, Artificial microRNA, Hypoxia, Abscisic acid signaling, *Arabidopsis thaliana*

## Abstract

**Background:**

Small proteins remain poorly characterized in *Arabidopsis thaliana*, partly because of genetic redundancy and the difficulty of identifying loss-of-function mutants. To address this gap, we investigated 58 hypoxia-responsive small protein genes (HRSPs) encoding proteins of 80–100 amino acids using a homology-based artificial microRNA (amiRNA) strategy.

**Results:**

Homozygous amiRNA lines were screened for developmental traits and stress-related phenotypes. Two closely related paralogs, HRSP41 and HRSP42, emerged as key regulators with both shared and distinct functions. HRSP41 promoted flowering under short-day conditions, whereas HRSP42 delayed flowering and specifically increased seed tolerance to oxidative stress. Both genes acted together to reduce the inhibitory effect of abscisic acid on seed germination. Transcriptome profiling revealed distinct downstream effects: HRSP42 silencing was associated with repression of glutathione metabolism, whereas HRSP41 silencing affected glycerolipid metabolism and cell wall-related pathways. Under hypoxia, simultaneous downregulation of HRSP41 and HRSP42 altered transcriptional, post-transcriptional, and translational responses and intensified the repression of photosynthesis-related genes.

**Conclusions:**

Despite their high sequence similarity, HRSP41 and HRSP42 control plant development and stress adaptation through partially overlapping but clearly distinct molecular mechanisms. Our results identify these paralogs as previously uncharacterized regulators of flowering, oxidative stress responses, ABA sensitivity, and hypoxia-responsive gene expression.

**Supplementary Information:**

The online version contains supplementary material available at https://doi.org/10.1186/s12870-026-09666-5.

## Introduction

Plants, as sessile organisms, rely on remarkable plasticity to survive environmental stress. To adapt, they deploy a wide array of proteins with structural, enzymatic, and signaling functions that enhance fitness and reprogram gene expression and metabolism under adverse conditions [1–3]. Despite extensive efforts using genome-wide omics approaches, a large fraction of plant proteins remains functionally uncharacterized. Transcriptomic profiling in *Arabidopsis thaliana* has suggested that approximately 20% of genes—nearly 5,000 out of 27,000—encode proteins of unknown function [4]. Strikingly, this percentage increases with decreasing protein size, and our preliminary analyses indicate that approximately half of Arabidopsis proteins ≤ 100 amino acids in length lack functional annotation. Although peptides and small proteins have been implicated in plant stress signaling and development [5], the roles of most of these molecules remain unknown.

The most recent Arabidopsis proteome annotation, Araport11, includes 39,346 proteins [6]. Although small proteins have gained research attention in the past decade [7–10], many of their functions remain elusive. T-DNA insertion libraries have accelerated functional genetics in Arabidopsis; however, ~ 12% of genes still lack insertions, and ~ 8% are represented by only a single allele [11]. Moreover, this approach faces two limitations: (i) lethality can prevent the recovery of null mutants [12], and (ii) high functional redundancy within gene families can mask phenotypes [13]. Additionally, short gene sequences are less likely to be disrupted by random T-DNA insertion.

Homology-based gene silencing via amiRNAs has emerged as a powerful alternative for functional plant genomics [14–17]. This computationally designed technology allows for the precise targeting of individual genes or simultaneous silencing of gene families, thereby addressing both redundancy and lethality. It can also generate an allelic series with graded expression knockdowns. Recently, cell type-specific amiRNA strategies have been applied to dissect the functions of essential genes in Arabidopsis [18].

In the context of climate change, plants face increasingly complex stress. Extreme temperatures and irregular rain patterns significantly threaten plant growth, with heavy rainfall often leading to flooding and oxygen deprivation in submerged tissues [19, 20]. While hypoxia-responsive pathways have been studied for their impact on physiology and metabolism [21, 22], the molecular roles of many proteins that sense and transduce oxygen limitation remain unknown. In particular, the contribution of small proteins to hypoxia and oxidative stress responses, both during oxygen deprivation and subsequent reoxygenation, remains poorly understood [23]. Hypoxia is a major environmental stress that reshapes plant physiology and gene expression. Although broad transcriptional responses to oxygen deprivation have been described, the functions of many hypoxia-responsive proteins, particularly small proteins, remain unknown. This gap is important because hypoxic stress often occurs together with oxidative stress and other physiological perturbations, and the proteins that integrate these responses are only partially understood.

amiRNAs provide a powerful alternative for functional analysis of small genes and redundant gene families [14–17]. By enabling targeted downregulation of one or more homologous transcripts, this approach can reveal phenotypes that may not be detectable through conventional mutant screens. It is therefore well suited to investigating small, poorly annotated genes whose disruption may be difficult or impossible using insertional mutagenesis.

Here, we focused on 58 hypoxia-responsive small protein genes, designated *HRSP* genes, encoding proteins of 80–100 amino acids. This set was selected to provide a collection large enough to rigorously evaluate our approach while remaining experimentally manageable within the scope of this study. We generated computationally designed amiRNA lines targeting individual HRSPs or closely related gene pairs and screened them for developmental and stress-related phenotypes. This strategy identified two paralogs, HRSP41 and HRSP42, as important regulators of flowering, vegetative development, oxidative stress tolerance, ABA-sensitive seed germination, and hypoxia-responsive gene expression. Despite their high sequence similarity, the two proteins showed both overlapping and distinct functions, providing new insight into the biological roles of plant small proteins.

## Materials and methods

### Plant growth conditions and stress tolerance and developmental assays

Arabidopsis (*Arabidopsis thaliana*) seeds were surface-sterilized with chlorine gas before being sown in MS (Duchefa Biochemie, The Netherlands) medium plates containing 1% (w/v) sucrose. Seeds from wild-type Col-0 and amiRNA-GUS were obtained from the Nottingham Arabidopsis Stock Center (NASC) and Alberto Carbonell [24], respectively. Col-0 plants were transformed by dipping floral organs in a suspension of transformed Agrobacterium tumefaciens C58C1 with the corresponding amiRNA constructs [25] and further selected on MS medium supplemented with 10 mg/L ammonium glufosinate (BASTA). Finally, independent lines were selected for the homozygous amiRNA plants that were 100% BASTA-resistant.

To test the tolerance to oxidative stress, seeds of the same age of the different genotypes were sown in water in 24-well plates and maintained under photoperiodic conditions (16 h light:8 h darkness; 100 µmol m^− 2^ s^− 1^) at 21 °C and supplemented with 10 µM paraquat/methyl viologen (MV) or without MV as control. Seed germination rates were calculated at the indicated times as the mean of three independent experiments, each with approximately 50 seeds per genotype and condition. To analyze foliar tolerance to MV, rosettes of 30-day were sprayed to run-off with 5 to 35 µM MV in water, and chlorosis damage was analyzed every 24 h up to 3 days after treatment. To analyze the sensitivity to ABA, seed germination assays were conducted as described above for oxidative stress but supplemented with MS medium with 0.25–0.5 µM ABA or no ABA addition as a control.

Flowering time was calculated as the number of leaves at bolting [26, 27] in plants grown under photoperiodic long-day (16 h light:8 h darkness) or short-day (8 h light:16 h darkness) conditions. Three independent experiments were performed by counting the leaves of 10 plants per analyzed genotype in each experiment.

Hypoxic conditions were generated by incubation in a box containing an inlet for N_2_ gas and an O_2_ sensor connected to a ProOx Model 110 controller that allowed for tight control of the O_2_ concentration inside the box (BioSpherix, NJ, USA). In vitro, 12-day-old plants grown under long-day photoperiodic conditions in MS medium supplemented with 1% (w/v) sucrose were exposed to hypoxia treatment in the dark at 1% (v/v) O_2_ for the indicated time.

### Generation of amiRNA plants

The design of amiRNA constructs targeting HRSP genes was computationally assisted using the P-SAMS amiRNA designer (https://p-sams.carringtonlab.org/) [17]. We designed and generated 62 amiRNAs targeting single genes or pair of closely related genes (Table S1). The 75-mer oligonucleotides specifically targeting each of the HRSP genes (Table S1) were annealed, and amiRNA sequences were cloned into the AtMIR390a-based pMDC123SB-AtMIR390a-B/c vector [16, 28]. The resulting plasmids were used to transform *Agrobacterium tumefaciens C58C1* strain, which was then used to genetically transform Arabidopsis plants by agroinfiltration using the dipping floral organ method [25]. Primary transformed plants resistant to ammonium glufosinate (BASTA) were either used for direct phenotypic screenings or grown to the T3 generation to obtain homozygous plants. We end up with a collection of 62 homozygous lines plus and additional one using and amiRNA against GUS reporter gene used as control wild-type line [24].

### RNA isolation, reverse transcription-quantitative PCR, and RNA-Seq analysis

RNA was extracted and purified using the Nucleospin RNA Plant kit (Macherey-Nagel, Germany), reverse transcribed with M-MuLV Reverse transcriptase (RNase H minus from Nzytech, Portugal) and oligo-dT, and the resulting cDNA was quantified by real-time PCR using an Applied Biosystems (USA) QuantStudioTM 3 PCR system with specific primer pairs (Table S2) for each HRSP gene, and the reference genes TIP41L (AT4G34270) and ACTIN2 (At3g18780) as previously reported [29, 30].

A genome-wide transcriptome analysis of 12 d-old wild type (Col-0) and amiRNA-HRSP plants, either grown under normoxic (21% O_2_) conditions or grown under normoxia and then subjected to hypoxia (1% O_2_) for 4 h, was performed by RNA-Seq (BGI Tech Solutions, Hong Kong). Three biologically independent RNA replicates were obtained for each genotype and condition. Libraries were prepared from oligo-dT-enriched mRNAs obtained from total RNAs with RIN > 8.0, as analyzed using a 2100 Bioanalyzer (Agilent Technologies Inc.). RNA was fragmented, and first-strand cDNA was generated using random N6-primed reverse transcription, followed by second-strand cDNA synthesis with dUTP instead of dTTP. The synthesized cDNAs were subjected to end-repair and then 3’ adenylated. Adaptors were ligated to the ends of these 3’ adenylated cDNA fragments, and prior to PCR amplification, the dUTP-marked strand was selectively degraded by uracil-DNA-glycosylase (UDG). The remaining strand was amplified to generate a cDNA library suitable for sequencing. After denaturing the PCR product by heat, the single-stranded DNA was cyclized by splint oligo and DNA ligase, DNA nanoball synthesis was performed, and finally, sequencing was performed using the DNBSEQ Technology platform. Raw data were filtered using SOAPnuke (v.1.5.2) software (with -l 15 -q 0.2 -n 0.05 parameters) developed by BGI. The filtered clean reads were saved in the FASTQ format. Reference genome alignment was performed using the Hierarchical Indexing for Spliced Alignment of Transcripts (HISTAT) software (v.2.0.4; Parameters:--sensitive --no-discordant --no-mixed -I 1 -X 1000 -p 8 --rna-strandness RF).

### In silico analyses and predictions

Gene Ontology analysis of functional category enrichment was performed using the ShinyGO 0.82 tool (https://bioinformatics.sdstate.edu/go/). In silico analysis of HRSP gene expression under different nutritional and stress treatments, as well as diverse developmental conditions, was conducted based on transcriptome data deposited in AtCAST3.1 (http://atpbasmd.yokohama-cu.ac.jp/cgi/atcast/search_input.cgi), Arabidopsis RNA-Seq Database (http://ipf.sustech.edu.cn/pub/athrna/), and Transcriptome Variation Analysis (TRAVA) database (https://travadb.org/). Gene list overlaps were analyzed using Venny 2.1.0 (https://bioinfogp.cnb.csic.es/tools/venny/index.html).

### Statistical analyses

The values of transcript levels, seed germination rates, and flowering time calculated as leaf numbers at bolting are presented as the mean of three independent biological replicates ± standard error (SE). Statistical significance was analyzed as indicated in the figure legends by unpaired Student´s t-test comparing any genotype to the wild type Col-0 values or any condition to the control, untreated set. Statistical significance is indicated by *p* < 0.05 (*), *p* < 0.005 (**), and *p* < 0.001 (***).

## Results

### Identification of hypoxia-responsive small protein genes

To identify small proteins that respond to low oxygen, we first analyzed *Arabidopsis thaliana* genes encoding proteins of 80–100 amino acids. This set comprised 998 proteins, representing approximately 2.5% of the predicted proteome, and nearly half were annotated as uncharacterized (Fig. 1A). We then compared this dataset with transcriptome profiles from wild-type plants exposed to hypoxia for 4 h [31;GSE214887] and identified 60 gene transcripts corresponding to 58 genes, because two of the genes were represented by two different transcripts, whose expression changed in response to low oxygen (Fig. 1A). These genes were designated *Hypoxia-Responsive Small Protein* genes, or *HRSP1* to *HRSP58*.

**Fig. 132:**
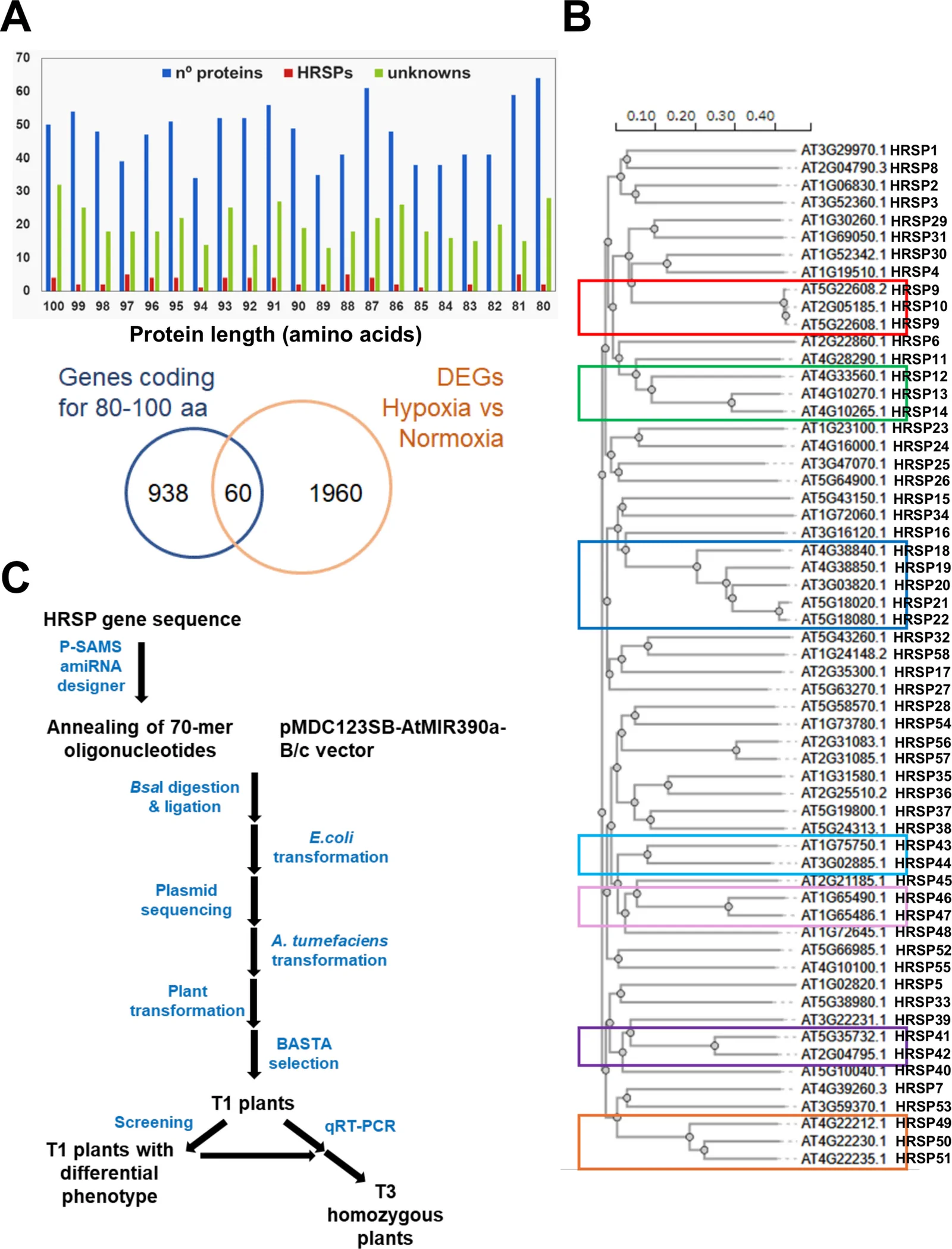
The Arabidopsis family of Hypoxia-Regulated Small Protein (HRSP)-encoding genes. **A** Number of proteins in the Arabidopsis proteome with unknown function and those encoded by *HRSP* genes in the size range of 80–100 amino acids. **B** Dendrogram showing the phylogenetic relationships between HRSP proteins. Clusters framed in different colors indicate potential paralogs. **C** Pipeline for the design and generation of amiRNAs, subsequent Arabidopsis transformation, and selection of homozygous lines.

The HRSP family was highly diverse in sequence and predicted function. Phylogenetic analysis grouped the proteins into distinct clusters (Fig. 1B), with some paralogs showing close similarity and suggesting possible functional redundancy. Because small loci are often difficult to disrupt by insertional mutagenesis, we used an amiRNA-based silencing strategy to target individual HRSP genes or closely related gene pairs. As summarized in Fig. 1c, amiRNAs were designed, annealed, and cloned into the binary vector pMDC123SB-AtMIR30a B/C, followed by transformation into Agrobacterium tumefaciens and subsequent transformation of Arabidopsis. Transgenic plants were selected using Basta and advanced to homozygosity in the T3 generation. This approach yielded 63 homozygous amiRNA lines for phenotypic analysis.

### In silico analysis of HRSPs

HRSP proteins are small (8.5–11.7 kDa), with 61% predicted to contain intrinsically disordered regions and 26% to be membrane-associated (Table S3). Transcriptome Variation Analysis (TRAVA) revealed broad expression diversity across organs and developmental stages (Table S4). While some HRSP genes were broadly expressed (e.g., HRSP7–10 and HRSP55), many displayed tissue-specific expression in reproductive organs, leaves, roots, siliques, the inflorescence meristem, or during senescence, suggesting diverse biological functions. Stress and hormone response datasets further demonstrated extensive regulatory diversity (Table S5). Most HRSP genes responded to multiple environmental, nutritional, or hormonal cues, particularly ethylene, ABA, salicylic acid, nutrient deprivation (P, S, and N), oxidative stress, and drought. A few genes exhibited more specific regulation, including HRSP7 and HRSP26 (phosphorus starvation), HRSP25 (ethylene repression), HRSP55 (ethylene induction), HRSP29 (ABA), and HRSP41 (NO).

### Phenotypes of amiR-HRSP lines

**Fig. 2:**
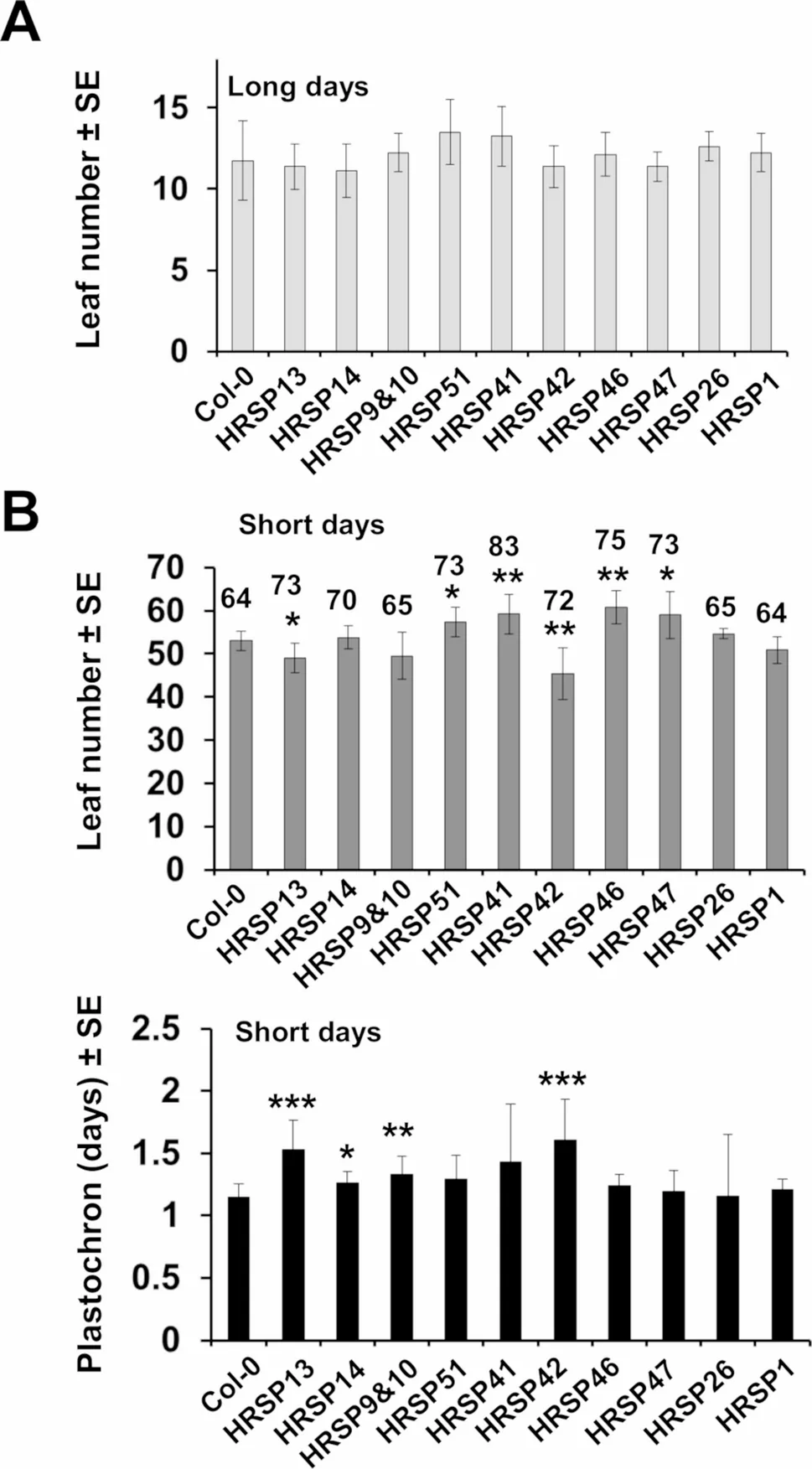
Flowering time of amiR-HRSP homozygous and wild-type plants grown under (**A**) long and (**B**) short days. Flowering time was calculated by counting the rosette leaves at bolting. Values are the means of three independent experiments, each with at least 10 individuals per genotype and condition. Error bars represent standard error (SE). The mean value of days from sowing to bolting is shown on top of each histogram. The bottom panel in (**B**) represents the plastochron for the indicated genotypes grown under short day conditions. Statistical analysis was performed using unpaired t´test to compare each genotype with Col-0 plants. *, **, *** represent p-values < 0.05, < 0.005 and < 0.001, respectively.

Because many *HRSP* genes were developmentally regulated and responsive to stress (Tables S4 and S5), we examined whether their silencing affected flowering time and oxidative stress tolerance. The analysis of flowering time under long-day conditions showed that most amiR-HRSP lines showed little or no deviation from wild type (Fig. 2A). Under short-day conditions, however, several lines displayed altered flowering time. amiR-HRSP13 and amiR-HRSP42 flowered earlier, whereas amiR-HRSP13, amiR-HRSP41, amiR-HRSP46, amiR-HRSP47, and amiR-HRSP51 flowered later than Col-0 (Fig. 2B). The early flowering phenotypes of amiR-HRSP13 and amiR-HRSP42 were accompanied by longer plastochrones (Fig. 2B).

**Fig. 3:**
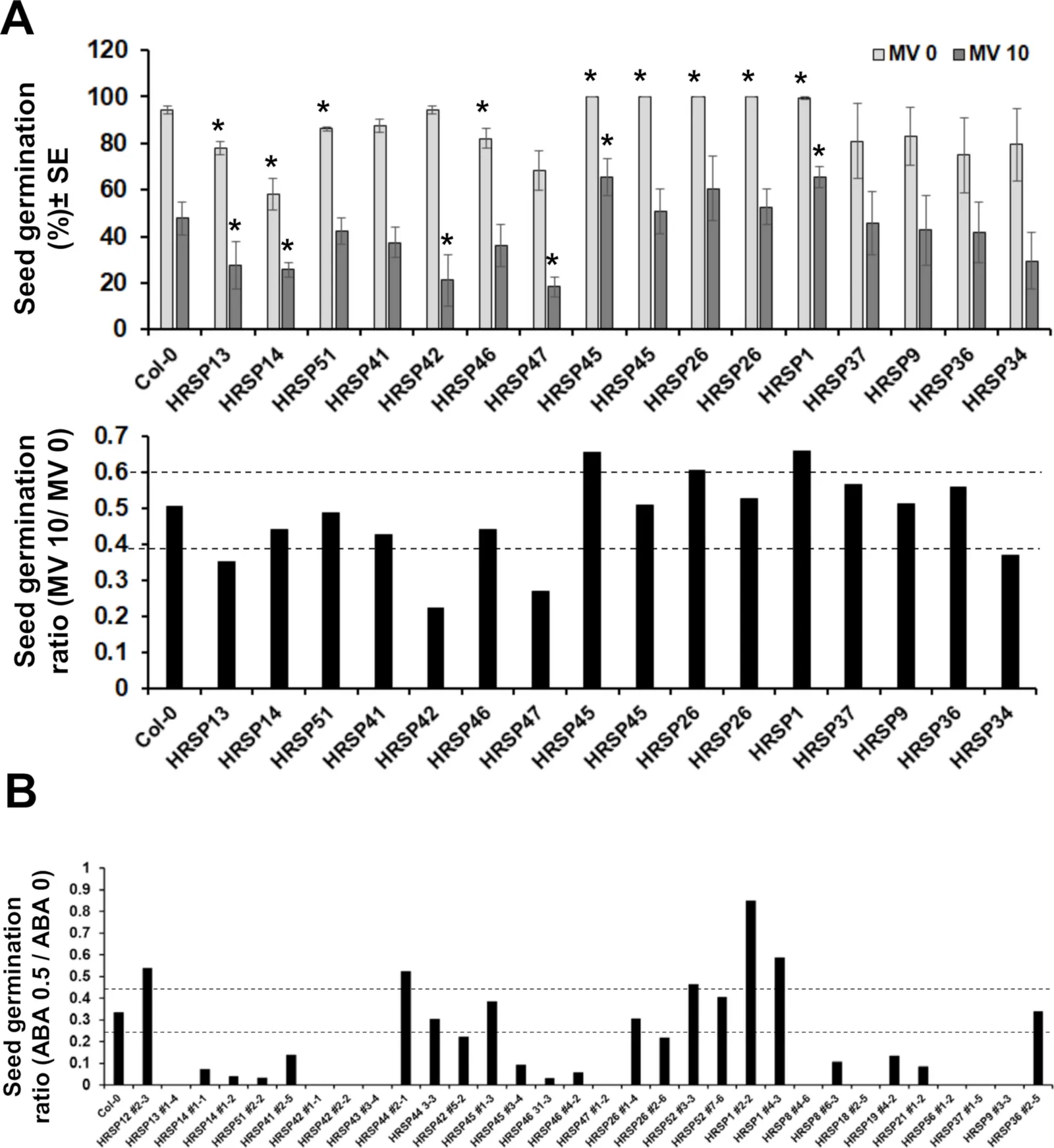
Effect of MV on seed germination of wild type and amiR-HRSP lines. (A) The values are the mean of three independent experiments, each with approximately 60 seeds per genotype and treatment. Error bars represent standard error (SE). Statistical analysis was performed using unpaired t´test comparing each genotype with Col-0 plants, either untreated or treated with 10 µM MV. *, ** represent p-values < 0.05, < 0.005, respectively. The bottom panel represents the ratio of seed germination in MV-treated versus untreated seeds. Dotted lines indicate variability limits in wild-type Col-0 seeds. (B) Ratio of seed germination in ABA-treated versus untreated seeds. Dotted lines indicate the variability limits in wild-type seeds.

As reactive oxygen species (ROS) are central to many stresses and developmental responses and regulate multiple HRSP genes, we tested whether *HRSP* knockdown affected tolerance to oxidative stress. Seeds of amiR-HRSP lines were germinated on MS medium supplemented with 10 µM MV or on control medium. As expected, all MV-treated seeds displayed reduced germination, but the responses varied among the lines (Fig. 3A). Two lines (amiR-HRSP1 and amiR-HRSP45) were less sensitive than the wild type, whereas three (amiR-HRSP42, amiR-HRSP47, and amiR-HRSP13) were more sensitive. Interestingly, the basal germination rates of untreated seeds also differed: amiR-HRSP13, -14, -47, and − 51 germinated more poorly than Col-0, whereas amiR-HRSP1, -26, and − 45 germinated slightly better. Because ABA is likely the most relevant factor regulating seed germination [32, 33], we also screened the amiRNA-HRSP lines in seed germination assays in the presence and absence of ABA. Figure 3B shows that, with some exceptions, most amiRNA-HRSP seeds were more sensitive than wild-type seeds to ABA. Seeds of amiRNA targeting HRSP8, 9, 13, 18, 37, 42, 43, 47, and 56 did not germinate, whereas more than 30% of wild-type seeds germinated at 0.5 µM ABA (Fig. 3B). In contrast, amiRNA-HRSP1, 12, and 44 germinated between 85% and 55% at 0.5 µM ABA (Fig. 3B). Basal germination in the absence of stress also varied among lines, indicating that some HRSPs may contribute to seed physiology under non-stressed conditions.

### HRSP41 and HRSP42 show divergent functions

Among the analyzed genes, *HRSP41* and *HRSP42* were especially notable because their corresponding amiRNA lines showed contrasting phenotypes (Figs. 2 and 3) despite the close similarity of the encoded proteins. The two encoded proteins are highly similar in amino acid sequence and predicted structure, with conserved N- and C-terminal α-helices and disordered loops, and appear to have arisen from recent gene duplication (Fig. S1A–C). BLASTP searches revealed homologs only in eudicots, but not in algae, bryophytes, ferns, gymnosperms, or monocots (Table S6), suggesting that these proteins arose late in evolution to perform new functions that are not known to date. No recognizable motifs were identified beyond the disordered regions.

**Fig. 4:**
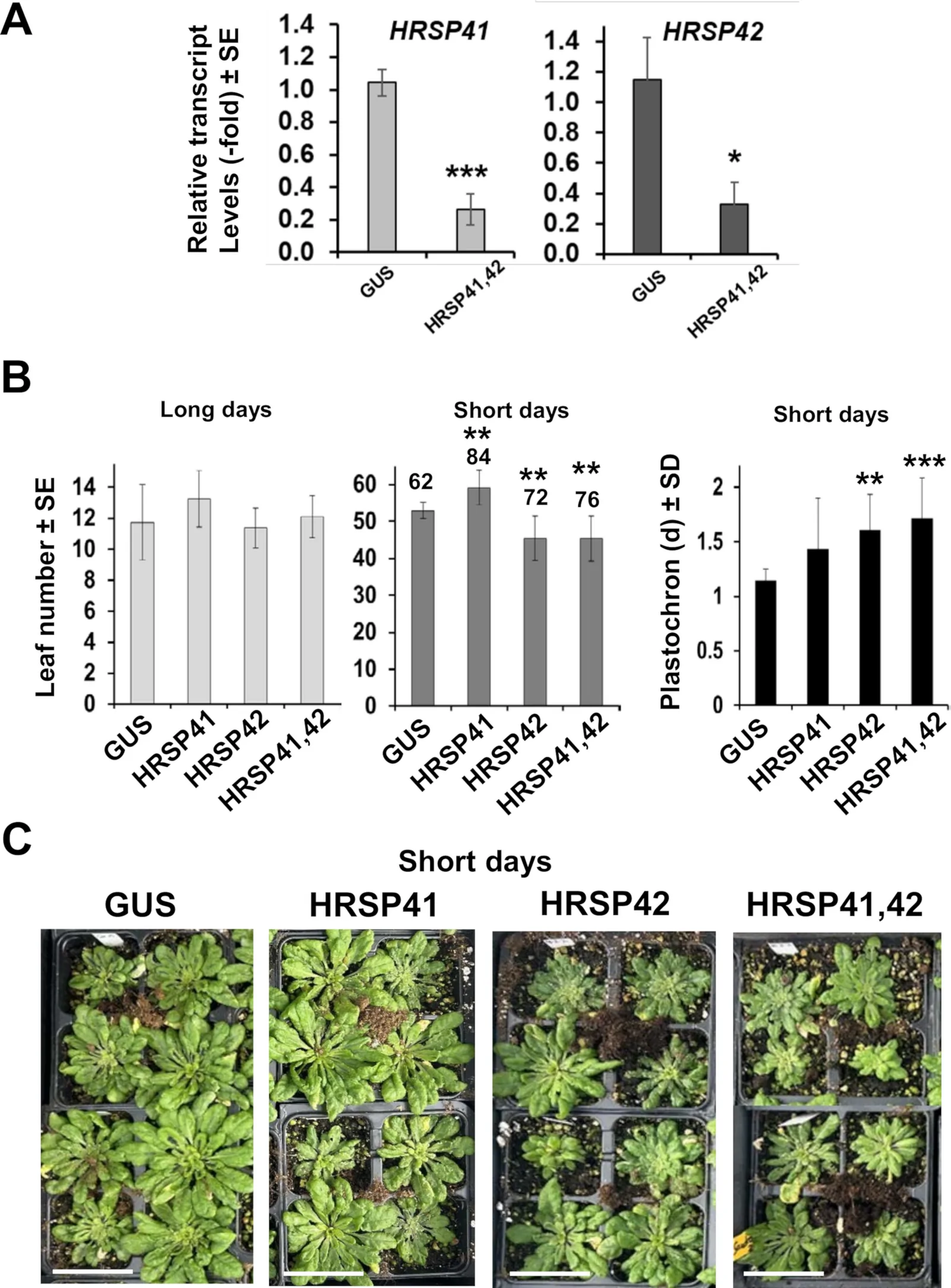
Flowering time phenotype of amiRNA-HRSP41, -HRSP42, and -HRSP41,42. **A** *HRSP41* and *HRSP42* transcript levels were quantified in amiR-GUS and amiR-HRSP41,42 seedlings by qPCR from three independent biological replicates. The mean values and SE are shown. Unpaired t-test was used for the statistical analysis. * *p* < 0.05 and *** *p* < 0.0005. **B** Flowering time was measured as the number of rosette leaves at bolting in plants grown under long or short days. Values are the means of three independent experiments, each with at least 10 individuals per genotype and condition. The mean value of days from sowing to bolting is shown on top of each histogram. Error bars represent standard error (SE). **C** Rosette phenotypes of short-day-grown plants of the indicated genotypes. Scale bars correspond to 5 cm.

To examine their functions in more detail, we analyzed single silencing lines for HRSP41 and HRSP42 and also generated a double silencing line targeting both genes (Fig. S1D) and compared to plants expressing an amiRNA targeting an exogenous gene (amiR-GUS) as a control [24]. In amiR-HRSP41,42 plants, the levels of *HRSP41* and *HRSP42* transcripts were around 30% of those detected in control plants (Fig. 4A). Expression patterns differed between the paralogs: HRSP41 was enriched in anthers and internodes, whereas both genes were strongly expressed in senescent leaves (Table S3). Phenotypic analysis revealed a clear functional divergence. Under long-day conditions, flowering time was unchanged (Fig. 4B). However, under short-day conditions amiR-HRSP42 and the double-silencing line flowered earlier with longer plastochrones, while amiR-HRSP41 flowered later than control plants (Fig. 4B). The early-flowering phenotype of the HRSP42-silenced line dominated in the double-silencing background, suggesting that HRSP42 reduction overrides the effect of HRSP41 silencing.

Vegetative growth showed a similar pattern. Under short days, amiR-HRSP42 and the double-silencing line formed smaller rosettes and leaves than either wild type or amiR-HRSP41 plants (Fig. 4C). These results indicate that HRSP41 and HRSP42 have both overlapping and distinct roles in developmental control, with HRSP42 acting as the dominant determinant in the combined silencing background.

### Transcriptome analysis of *HRSP41* and *HRSP42* silencing

**Fig. 5:**
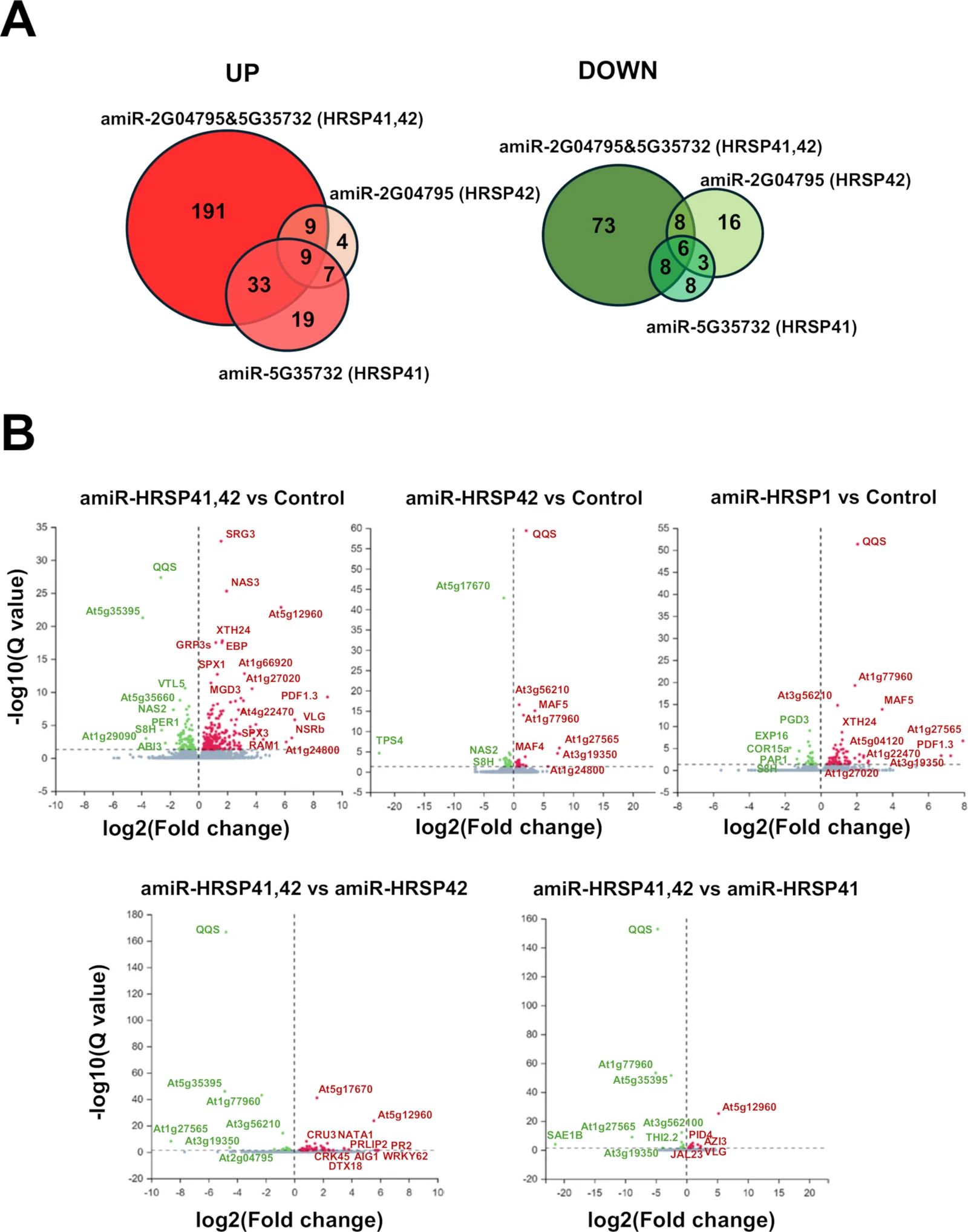
RNA-Seq analysis of amiRNA-HRSP41, -HRSP42, and -HRSP41&42 plants. **A** Venn diagrams showing the intersection between all three amiRNA genotypes for upregulated (red, left panel) and downregulated (green, right panel) DEGs. **B** Volcano plots showing the distribution of DEGs identified in any of the comparisons indicated at the top of each panel.

To investigate functional divergence between HRSP41 and HRSP42, we performed RNA-seq on amiR-HRSP41, amiR-HRSP42, amiR-HRSP41,42, and amiR-GUS control plants. Silencing HRSP41 or HRSP42 individually caused relatively modest transcriptional changes (93 and 62 DEGs, respectively), whereas the double-silencing line exhibited a much stronger response (337 DEGs; Fig. 5A, B; Tables S7–S9), suggesting partial functional redundancy. Functional enrichment analysis revealed both shared and distinct regulatory roles. HRSP41 silencing primarily affected glycerophospholipid metabolism, cell wall organization, photosynthesis, and stress responses, whereas HRSP42 silencing preferentially altered MAPK signaling, ABA responses, glutathione metabolism, and abiotic stress pathways. The double-silencing line showed broader effects on lipid, nitrogen, sulfur, and carbon metabolism, photosynthesis, and glutathione metabolism. Repression of the pentose phosphate pathway was common to all genotypes, while glycerolipid metabolism and glutathione metabolism were specifically shared between HRSP41/double-silencing and HRSP42/double-silencing plants, respectively. Analysis of genotype-specific DEGs further supported partial redundancy, with the double-silencing line displaying far more unique DEGs than either single mutant and enrichment for phosphate starvation, carbon metabolism, transport, desiccation, and glucosinolate-related processes (Fig. 5A; Table S10).

### Response to hypoxia

**Fig. 6:**
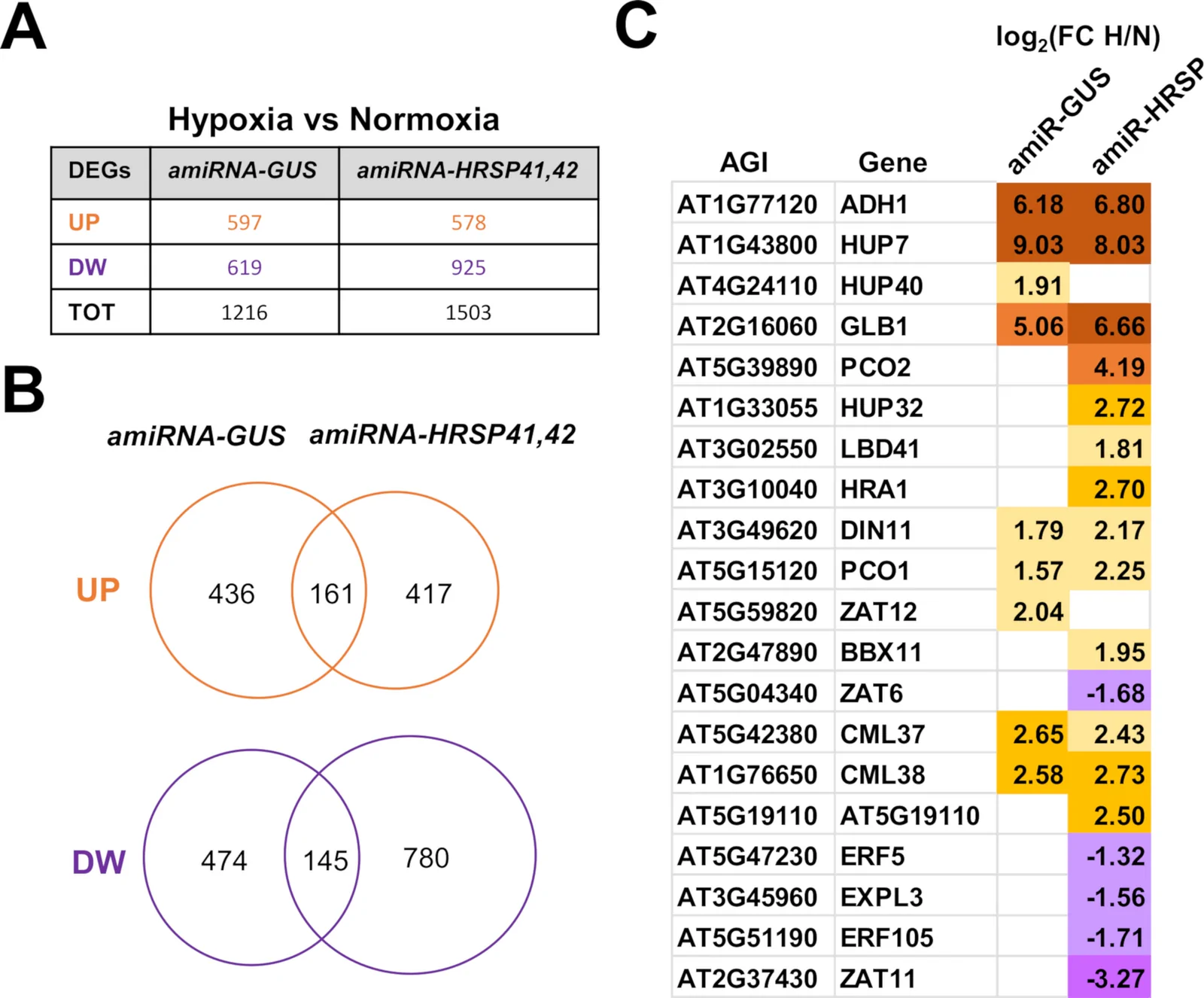
Hypoxia-responsive transcriptome of amiRNA-HRSP41,42 and amiRNA-GUS control plants. **A** Number of upregulated, downregulated and total DEGs identified when compared hypoxic to normoxic plants of each genotype. **B** Venn diagrams showing the common and distinct DEGs identified in hypoxic versus normoxic plants of the indicated genotypes. **C** Hypoxia-triggered regulation of genes of the core hypoxia response in Arabidopsis.

Because HRSP41 and HRSP42 are hypoxia-responsive genes, we examined whether they contribute to transcriptomic reprogramming during hypoxia. RNA-Seq of amiR-HRSP41,42 plants and amiR-GUS plants under hypoxia and normoxia was performed and both conditions compared for each genotype (Tables S11 and S12). The total number of DEGs, 1216 (597 upregulated and 619 downregulated) and 1503 (578 upregulated and 925 downregulated), for control and amiR-HRSP41,42 plants, respectively, were similar for both genotypes (Fig. 6A). However, only a fraction of DEGs (161 upregulated and 145 downregulated) were common in the hypoxia response of both genotypes (Fig. 6B), thus suggesting the transcriptome reprogramming triggered by hypoxia was mostly different in amiR-HRSP41,42 and control plants. In fact, several genes reported to be in the core of Arabidopsis response to hypoxia were significantly different in both genotypes (Fig. 6C) pointing to a role of HRSP41 and HRSP42 in setting up part of the transcriptional responses during hypoxia. When the transcriptome of hypoxic amiR-HRSP41,42 plants was compared to that of hypoxic control plants, 362 upregulated and 403 downregulated DEGs were identified (Table S13). The upregulated gene set showed limited functional enrichment restricted to indole-3-acetic acid amido synthetase activity thus suggesting a specific involvement of HRSP41 and HRSP42 in auxin-related metabolism (Table S13). Downregulated DEGs were strongly enriched in photosynthesis, translation, RNA binding, and metabolic processes (Table S13). Together these findings suggest that HRSP41 and HRSP42 are required for the full transcriptional response to hypoxia, particularly enforcing the usual suppression of photosynthesis-related functions under low oxygen. The results also indicate that the two paralogs participate in both shared and distinct branches of the hypoxic response.

### Oxidative stress and ABA sensitivity

**Fig. 7:**
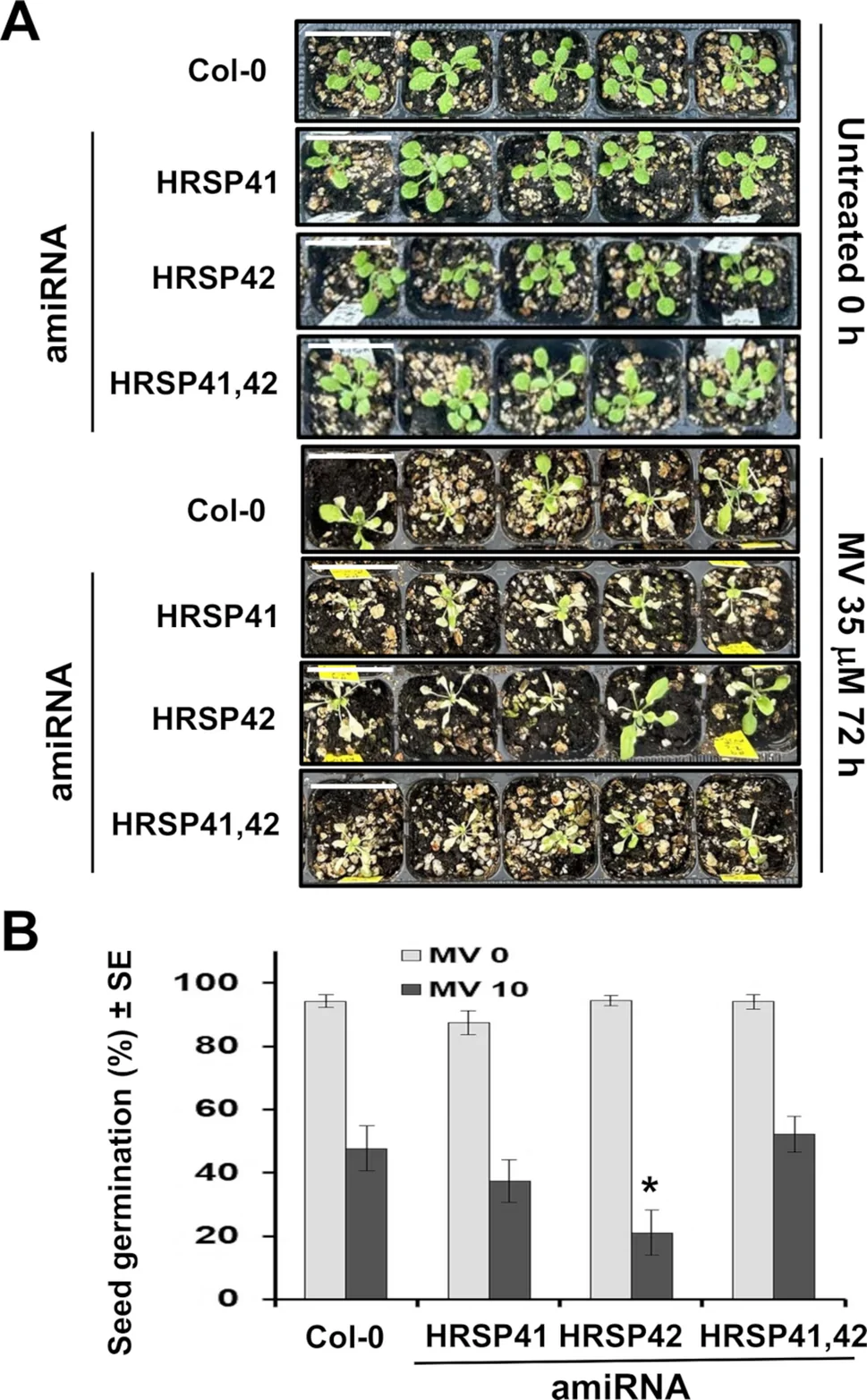
Effect of MV-triggered oxidative stress on (**A**) leaves and (**B**) seeds of wild-type and amiRNA plants targeting HRSP41 and HRSP42. The values in (**B**) are the means of three independent experiments, each with approximately 60 seeds per genotype and treatment. Error bars represent standard error (SE). Statistical analysis was performed using unpaired t´test comparing each genotype with Col-0 plants, either untreated or treated with 10 µM MV. * represents p-value < 0.05. Scale bars correspond to 5 cm.

Transcriptome analysis suggested that HRSP41 and HRSP42 may also contribute to oxidative stress responses. We therefore tested seed and vegetative tolerance to oxidative stress using MV. For foliar assays, we first optimized MV treatments in Col-0 plants by testing concentrations from 15 to 35 µM and exposure times between 24 and 72 h. A regime of 35 µM MV for 72 h was chosen, as it consistently caused bleaching and reduced rosette size in wild-type plants (Figure S2). All HRSP-silenced lines were more sensitive than wild type in foliar assays, with the strongest damage in amiR-HRSP41 and the double-silencing line (Fig. 7A). In contrast, seed germination assays showed that treatment with 10 µM MV reduced control wild-type germination by ~ 50% and seeds of amiR-HRSP41 and amiR-HRSP41,42 plants displayed similar levels of tolerance (Fig. 7B). In contrast, amiR-HRSP42 seeds were significantly more sensitive, showing only ~ 20% germination under MV treatment (Fig. 7B). These results indicate that HRSP41 and HRSP42 contribute to oxidative stress tolerance in a tissue-dependent manner. HRSP41 appears to be more important in vegetative tissues, whereas HRSP42 is more critical for maintaining seed germination under oxidative stress.

**Fig. 8:**
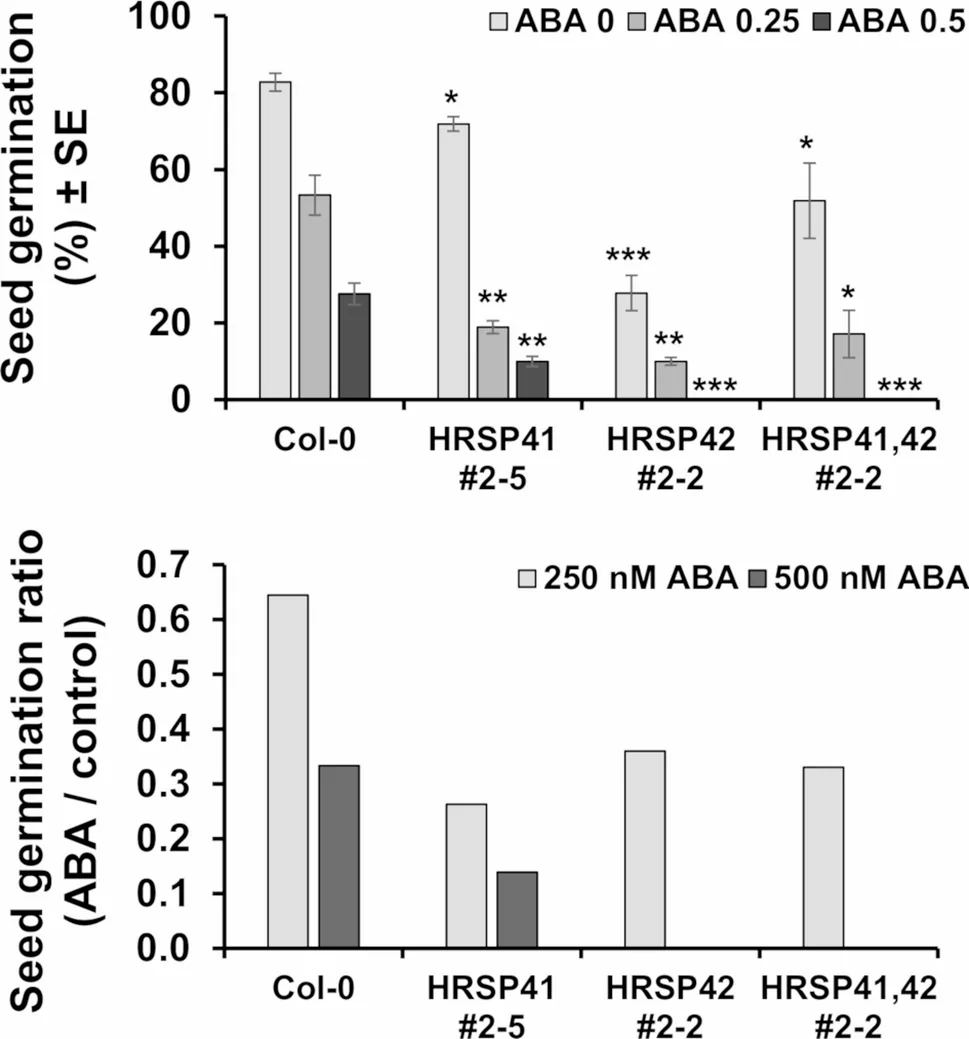
Effect of ABA on wild type and amiRNA-HRSP41, amiRNA-HRSP42, and amiRNA-HRSP41,42 seed germination. The upper panel shows the seed germination percentages as the mean of three independent experiments, each with approximately 60 seeds per genotype and treatment. Error bars represent standard error (SE). Statistical analysis was performed using unpaired t´test comparing each genotype with Col-0 plants, either untreated or treated with the indicated concentrations of ABA. *, **, and *** represent p-values < 0.05, < 0,005, and < 0.001, respectively. (b) The bottom panel shows the germination ratio of ABA-treated versus untreated seeds at the indicated ABA concentrations

ABA assays on seed germination supported this functional divergence. Silencing of HRSP41 or HRSP42 increased sensitivity to ABA during germination, and the effect was strongest when HRSP42 was silenced alone or together with HRSP41. At 0.5 µM ABA, wild-type seeds still germinated, but germination was strongly reduced in amiR-HRSP41 seeds and abolished in the HRSP42-silenced backgrounds (Fig. 8). These findings suggest that HRSP41 and HRSP42 cooperate to counteract ABA-mediated inhibition of seed germination, with HRSP42 making the dominant contribution.

## Discussion

This study shows that a class of previously uncharacterized hypoxia-responsive small proteins contributes to both development and stress adaptation in *Arabidopsis thaliana*. By using an amiRNA-based screening strategy, we were able to overcome a major limitation of functional studies on small genes, namely the difficulty of recovering informative loss-of-function alleles by insertional T-DNA mutagenesis. For such loci, computer-assisted amiRNAs designed to target specific sequences offer a valuable alternative [17, 34]. Importantly, amiRNAs can be designed to target single or multiple homologous genes, and by causing transcriptional downregulation or translational inhibition rather than full knockout [35], they are especially useful for studying redundant or essential genes, the complete loss of which may be lethal. This approach allowed the identification of HRSP41 and HRSP42 as paralogs with overlapping but clearly non-redundant roles.

A central finding is that HRSP41 and HRSP42 regulate flowering and vegetative growth in a short-day-specific manner. Under these conditions, HRSP41 silencing delayed flowering, whereas HRSP42 silencing accelerated it, and the double-silencing line followed the HRSP42 phenotype. This suggests that the two proteins act within a shared developmental framework but do not contribute equally to the same output. The effect of HRSP42 in the double-silencing background suggest that HRSP42 may be the dominant determinant of this trait when both transcripts are reduced. The short-day specificity also suggests that these proteins may intersect with specific flowering pathways such as the autonomous/vernalization pathway rather than with the general flowering machinery.

The transcriptome data provide additional support for this interpretation. Accordingly, we identified the K-box region and MADS-box transcription factors AGL68/MAF5 and AGL69/MAF4 encoding genes among the upregulated DEGs in amiRNA-HRSP41 and amiRNA-HRSP42 transcriptomes, but only the latter also upregulated the AGL9/SEP3 gene (Table S7, S8). In the amiRNA-HRSP41,42 differential transcriptome, we found that AGL68/MAF5 and AGL9/SEP3, but not AGL69/MAF4, were upregulated. MAF4 and MAF5 have been reported to play opposite roles in vernalization-triggered flowering in Arabidopsis [36]. SEP3 appears to mediate protein complex formation, which is important not only for regulating floral organ identity specification but also for regulating flowering time [37]. Moreover, HRSP41- and HRSP42-silenced lines showed distinct enrichment patterns, including altered expression of genes linked to hormone responses, cell wall organization, lipid metabolism, and stress signaling. In particular, the association of HRSP41 with glycerophospholipid and cell wall-related pathways suggests a role in developmental remodeling, whereas the HRSP42-associated repression of glutathione metabolism points to a stronger connection with redox homeostasis. The overlap in downregulation of the pentose phosphate pathway across genotypes is notable because this pathway contributes to cellular reducing power, reinforcing the idea that these HRSP proteins affect metabolic routes that support oxidative stress adaptation.

The differential stress phenotypes further indicate that HRSP41 and HRSP42 are not simply interchangeable. HRSP42 had a stronger role in seed germination under oxidative stress or ABA treatment, while HRSP41 appeared more important for vegetative tolerance to MV. This tissue-specific behavior may reflect distinct expression domains, different levels of residual transcript after silencing, or divergent biochemical functions. The stronger ABA hypersensitivity observed in the HRSP42 background also fits with the transcriptomic enrichment of ABA-related categories and supports a role for HRSP42 in buffering germination against inhibitory hormonal signals.

An important aspect of this work is the hypoxia response. Although both genes are hypoxia responsive, silencing them did not merely weaken one stress pathway; instead, it altered the broader transcriptional program induced by low oxygen. The strongest effect was seen in the double-silencing line, where genes associated with photosynthesis, translation, RNA binding, and metabolism were repressed. This suggests that HRSP41 and HRSP42 contribute to the maintenance of gene-expression control during hypoxia, potentially acting at transcriptional, post-transcriptional, or translational levels. The enhanced repression of photosynthesis-related genes is also consistent with a role in coordinating the energetic remodeling that accompanies oxygen deprivation. Nevertheless, the biological significance of HRSP41 and HRSP42 may extend beyond hypoxia itself. Their induction by multiple stresses and their involvement in ABA- and oxidative stress-related phenotypes suggest that they function as integrative regulators linking developmental transitions with environmental response pathways. Small proteins are increasingly recognized as important signaling and regulatory components in plants, and this study adds two further examples that act in complex, context-dependent ways. The fact that related homologs were found only in eudicots also raises the possibility that these proteins evolved relatively recently to support lineage-specific regulatory functions.

The amiRNA approach for functional characterization has, however, some limitations. Because amiRNA-mediated silencing does not completely eliminate transcript function, the phenotypes observed here likely reflect partial knockdown rather than null mutations. In addition, some apparent differences between genotypes may be influenced by tissue-specific silencing efficiency or by residual expression of the targeted genes. Future work using independent genetic approaches, protein localization studies, and biochemical interaction analyses will be needed to define the molecular mechanisms by which HRSP41 and HRSP42 act.

Overall, this work demonstrates that HRSP41 and HRSP42 are important regulators of flowering, vegetative growth, ABA sensitivity, oxidative stress tolerance, and hypoxia-responsive transcriptional reprogramming. More broadly, it shows that amiRNA-based functional screening is an effective strategy for uncovering the roles of small and redundant plant genes that are otherwise difficult to study.

## Conclusions

HRSP41 and HRSP42 are hypoxia-responsive small proteins with partially overlapping but distinct contributions to Arabidopsis development and stress responses. Our results suggest that HRSP41 is more closely associated with vegetative growth and the transition to flowering, whereas HRSP42 may contribute more prominently to ABA sensitivity, seed germination, oxidative stress tolerance, and transcriptional responses under hypoxia. Overall, these findings provide evidence that HRSP41 and HRSP42 participate in the coordination of developmental and environmental response pathways and establish a foundation for future studies on the functions of this small-protein family.

## Supplementary Information

Supplementary Material 1.

Supplementary Material 2.

Supplementary Material 3.

## Data Availability

The data supporting the findings of this study are included in the article and Supplementary Information files. RNA-Seq data have been deposited in the GEO database under accession numbers GSE214887 and GSE308033.

## Abbreviations

amiRNA: Artificial microRNA
DEG: Differentially expressed gene
HRSP: Hypoxia-responsive small protein
M-MuLV: Moloney Murine Leukemia Virus
RNA-Seq: RNA sequencing
